# 

**Published:** 1889-07

**Authors:** 


					﻿vol. ix.
JULY, 1889.
No. 7.
THE
HOMEOPATHIC pHYjSlClAfl,
A Monthly Journal of Homoeopathic Materia Medica
and Clinical Medicine.
“•He is a freeman whom truth makes free, and all are slaves beside.”
“Seek the Truth: come whence it may, cost what it will" ■	»
EDITED AND PUBLISHED BY
Edmund j. lee, mlid., .
AND
XVALTER ML JAMES, M. ID.
PHILADELPHIA :
No. 1183 SEURTTC® 8TR1D1DT.
LONDON
ALFRED HE^.TH <fc CO., Sole Agents foe Great Britain,
114 Ebury Street, Eaton Square.
’-Yearly Subscription, $2.50, invariably in advance. Single numbers, 25 cts.
Single numbers containing the Repertory Supplement, 50 cts.
Entered at the Philadelphia Poet-Office aa Second-Claas Mall Matter.
				

